# Exploring the potential malleability of spatial skills through anatomy teaching: A quantitative study among medical students

**DOI:** 10.1002/ase.70071

**Published:** 2025-06-13

**Authors:** Mandeep Gill Sagoo, Pak Yin Lam, Sharukesi Theivendran, Richard Wingate

**Affiliations:** ^1^ Faculty of Life Sciences and Medicine King's College London London UK

**Keywords:** anatomy teaching, cross‐sections, medical student, spatial skills

## Abstract

Spatial skills, or spatial ability, is the ability to visualize and mentally manipulate three‐dimensional objects, and is essential to the study of anatomy. This study aims to investigate whether spatial skills required to infer cross‐sectional images could be improved through anatomy learning, as well as gender differences in spatial skills. First‐year medical students from 2016, 2018, and 2019 at King's College London undertook two online tests examining their ability to identify cross‐sections of various anatomical structures and geometric solids from the Santa Barbara Solids Test (SBST). Test 1 took place in October before anatomy teaching, and Test 2 in December after anatomy teaching on the abdomen and pelvis. Mean scores were compared between Test 1 and Test 2 using the paired *t*‐test, and between male and female students using the chi‐squared test. Results demonstrate a trend toward improved performance in anatomical questions following anatomy teaching, although the extent of improvement was not consistent across cohorts. This trend remained evident even after exclusion of questions on the abdomen and pelvis. However, mean scores for SBST questions decreased significantly. No significant difference in spatial performance was observed between males and females. Our results suggest that spatial reasoning of anatomical cross‐sections could potentially be improved through anatomy teaching. Furthermore, improvements in spatial ability may be transferrable across body systems through the development and activation of mental schemas. However, they may not be transferrable to the broader domain of geometric solids, necessitating further research.

## INTRODUCTION

Spatial skills, or spatial ability, is the ability to apprehend, encode, and mentally manipulate objects in three dimensions.^1^ This encompasses *spatial perception*, identifying spatial positions in relation to other objects; *mental rotation*, viewing three‐dimensional (3D) objects from different angles and perspectives; and *spatial visualization*, a combination of multi‐step mental manipulations involving both spatial perception and mental rotation.^2^ Spatial skills are integral to the study of anatomy, which requires visualization of 3D anatomical structures, understanding their forms, shapes, and relationships within the body.^3^, ^4^ A systematic review by Langlois et al.^5^ identified a significant relationship between spatial performance and practical anatomy scores, while Lufler et al. reported that students who scored in the top quartile in spatial skills performed better in their final assessments in anatomy.^6^ At higher levels of clinical training, spatial performance is associated with a higher rate of skill acquisition among trainee surgeons, particularly in laparoscopy, key‐hole surgery, and robotic surgery.^7^, ^8^, ^9^

A fundamental feature of anatomy learning is the ability to interpret cross‐sectional images: to visualize, section, and rotate two‐dimensional (2D) slices of 3D objects along different planes. This is a complex task that involves all three facets of spatial ability as previously described, yet is central to many aspects of clinical practice, particularly in the analysis and inference of medical images such as x‐ray scans, computed tomography (CT) and magnetic resonance imaging (MRI).^10^, ^11^, ^12^ The positive correlation between spatial performance and the ability to interpret cross‐sectional images is widely reported in the literature, with evidence of its impact on performance in biological sciences and medicine.^13^, ^14^, ^15^

However, not all individuals are equally well‐equipped with spatial abilities; significant differences in spatial skills have been identified among high school and university students.^13^, ^14^, ^16^, ^17^, ^18^, ^19^ This raises the crucial question: are spatial skills wholly innate, or can they be improved over time? While it is believed that recognition of everyday objects and images, known as *System 1* thinking, is spontaneous and innate,^20^, ^21^ a growing body of evidence suggests that the ability to mentally manipulate complex images, or *System 2* thinking, can be facilitated and enhanced with relevant training.^22^, ^23^ Recent studies have explored a range of pedagogical techniques for improving spatial skills to enhance learning across the fields of science, technology, engineering, and mathematics (STEM),^22^, ^23^, ^24^, ^25^ incorporating physical models (active manipulation) as well as virtual models and animations (passive viewing). Duesbury and O'Neil used manipulable computer‐generated models to train engineering students to infer 3D dimensional objects from their 2D projections.^26^ Similarly, Cohen and Hegarty introduced a training protocol utilizing virtual models and animations, significantly improving undergraduate students' spatial ability at identifying cross‐sections of standard 3D geometric solids such as cones, cubes, cylinders, prisms, and pyramids.^27^

In anatomy education, studies have posited that spatial ability could be enhanced through dissections and prosections.^28^, ^29^, ^30^, ^31^ Other teaching modalities such as 3D printing, virtual models, augmented reality, and body painting have also demonstrated a potential positive impact on spatial skills.^32^, ^33^, ^34^ However, there remains a paucity of studies investigating the influence of anatomy education on the ability to infer cross‐sectional images specifically.

This study hypothesizes that spatial skills in the analysis of anatomical cross‐sectional images can be improved through anatomy education over time. This may be supported by existing cognitive theories. Piaget's Schema theory proposes that individuals organize information through the construction of *schemas*: internal mental frameworks that continuously evolve to incorporate new experiences through the processes of assimilation and accommodation, and consequently influence the individual's ability to interpret novel concepts based on past experiences.^35^ In this manner, during anatomical teaching, students begin to form schemas based upon knowledge of anatomical structures and their spatial relationships. The development of mental schemas may be enhanced by the integration of classroom‐based and practical approaches in anatomy teaching, as supported by Paivio's Dual Coding theory,^36^ which posits that individuals process information through both verbal and visual means. These systems operate independently but contemporaneously, interacting with each other to generate multiple pathways of information recall, memory retention, and assimilation and interpretation of novel concepts, facilitating improvements in spatial performance over time.

Additionally, studies exploring the association between gender and spatial ability have reported mixed findings. Earliest studies date back to Maccoby and Jacklin,^37^ who demonstrated that males outperform females in spatial skills.^37^ Subsequent studies and meta‐analyses have reported that males score higher in spatial skills tests than females, particularly in mental rotation tasks.^38^, ^39^, ^40^, ^41^, ^42^ In a group of medical graduates entering residency training, Langlois et al.^43^ found that male graduates outperformed their female counterparts in spatial performance. Similarly, Sarilita et al.^44^ in a cross‐sectional study of dental students reported that male students scored significantly higher in both the Vandenberg and Kuse Mental Rotation Test and the Revised Purdue Spatial Visualization Test. Several studies have attributed this gender disparity to both innate biological differences between males and females^45^, ^46^ and environmental influences, such as schooling and childhood activities.^47^, ^48^ The meta‐analysis by Baenninger and Newcombe^49^ suggested that both males and females hold equal potential at improving their spatial skills through training regardless of whether one outperforms the other initially.^49^, ^50^, ^51^ More recently, Gold et al.^52^ reported that while male undergraduate students significantly outperformed female students on mental rotation tasks, this disparity was fully mediated after adjusting for academic performance as well as childhood extracurricular activities involving spatial reasoning such as action, construction, or sports video games. This further suggests that gender differences in spatial skills may not be fully biologically innate and could potentially be improved through cumulative experience and training over time.

This study has two objectives: first, to investigate the hypothesis that students' spatial ability to interpret 2D cross‐sectional images, including anatomical regions and geometrical objects, could be improved through anatomy education over time; and second, to explore whether there exist any differences in spatial performance between male and female students. To this end, online multiple‐choice tests were designed and administered to first‐year medical students to assess their spatial skills in identifying cross‐sections of anatomical and geometrical structures, before and after two months of anatomy teaching. Results were compared to see whether there is an improvement in test scores after anatomy teaching and to evaluate any differences in scores between male and female students.

## METHODS

### Participant demographics

This study was repeated three times (2016, 2018, 2019), involving a total of 188 first‐year medical students: 88 students in 2016, 52 students in 2018, and 49 students in 2019. The proportion of male vs. female students in each cohort is presented in Table 1. In all cohorts, there were more female students than male students.

**TABLE 1:** Proportion of male vs. female students in each cohort.

Cohort	Total participants	Male	Female
2016	88	26 (29.5%)	62 (70.5%)
2018	52	14 (26.9%)	38 (73.1%)
2019	49	15 (30.6%)	34 (69.4%)

### Design of spatial skills tests

Two bespoke online tests of multiple‐choice questions were used to assess students' spatial skills before and after anatomy teaching. Each question consisted of an image of either an anatomical structure or geometric 3D object, with a superimposed plane along which the object is to be sliced. The task was to choose the correct cross‐sectional image from one of four possible choices.

**FIGURE 1:**
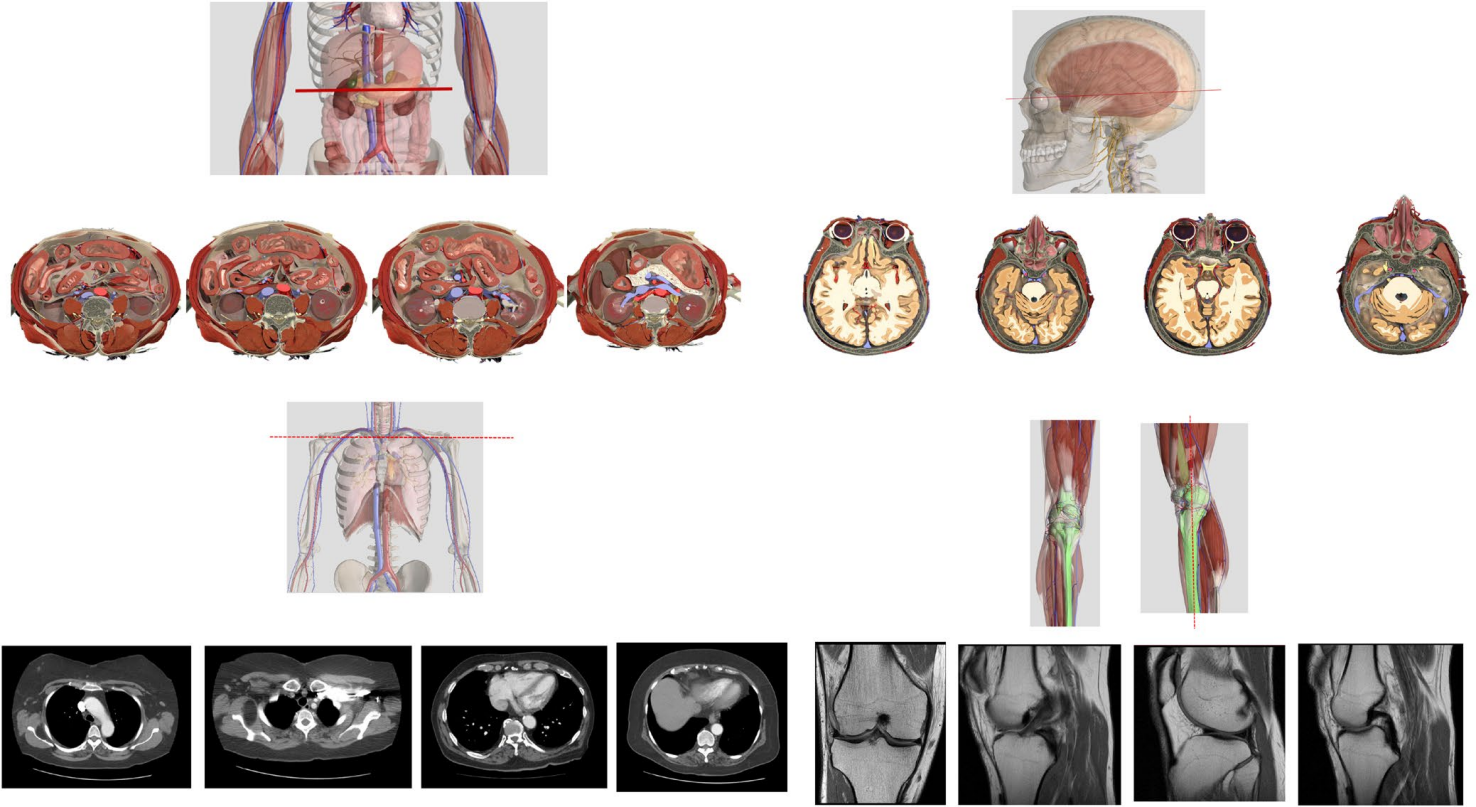
Sample anatomical questions created using Complete Anatomy Lab. Students are given an image of an anatomical structure with a superimposed plane (red line) along which the structure is to be sliced. Students are tasked to choose the correct cross‐sectional image out of 4 choices.

**FIGURE 2:**
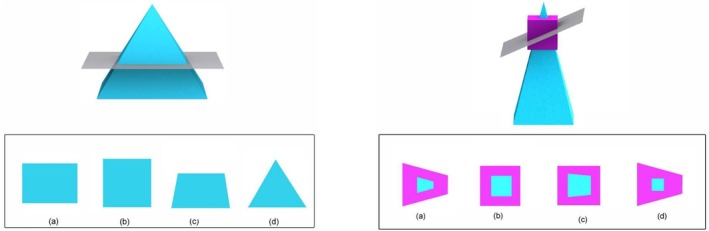
Sample geometric solids questions using 3D models from the Santa Barbara Solids Test.

Anatomical images were created using Complete Anatomy Lab (3D4 Medical, Elsevier, Amsterdam, Netherlands) (Figure 1). These included 3D models of anatomical structures and radiological images. The software included features that allowed specific structures to be hidden, as well as superficial structures to be made translucent, highlighted, labeled, and cut, enabling the design of highly customized images for each question. Standard geometric 3D objects were obtained from the Santa Barbara Solids Test (SBST), a widely recognized test of spatial ability first introduced by Cohen and Hegarty,^13^ and later validated and used among college students across many STEM disciplines. Three types of geometric SBST figures were included: *simple*, *joined*, and *embedded* figures. Simple figures include the primitive geometric solids: prisms, pyramids, cylinders, and cones (Figure 2A). Joined figures consist of two simple figures attached by their edges, while embedded figures consist of a simple figure enmeshed inside another (Figure 2B).^13^

Each test was comprised of 12 anatomical questions (3 relating to the abdomen and pelvis, plus 9 on other body systems) and 12 SBST questions, yielding a total of 24 questions. Cronbach's alpha was calculated for each test to evaluate the internal consistency of student responses.

### Delivery of spatial skills tests and anatomy teaching

First‐year medical students at King's College London in cohorts 2016, 2018 and 2019 participated in the study. The first test (Test 1) was delivered in October at the start of the school year. During the first school term, students received anatomy teaching as part of the *Functional Anatomy and Embryology* module, which ran from September to December. Teaching consisted of lectures, small‐group workshops and weekly dissections. Details of the curriculum, and time spent per week on each learning activity, are listed in Tables S1 and S2. In accordance with the curriculum, anatomy teaching during October to November mainly focused on the abdomen and pelvis. While there was no special emphasis on cross‐sectional anatomy in the curriculum, medical images such as cross‐sectional CT and MRI scans were used occasionally during lectures. Additionally, online learning tools such as Acland's Anatomy (Wolters Kluwer, Netherlands) and An@tomedia (Melbourne, Australia) offered students an extensive range of both virtual and physical anatomical models for learning. Nevertheless, these online materials were for optional self‐directed learning only and did not constitute any mandatory timetabled activities. Students then undertook the second test (Test 2) in December. Both tests contained the same number of questions and were of similar difficulty.

The tests were designed to be optional and formative in nature, meaning they do not impact students' academic grades. The tests were designed and collated by MGS, and delivered using the university's intranet, where students had their own accounts and unique login credentials for access. Prior to starting each test, written instructions were provided to contextualize the questions and provide visual aids. The use of an online multiple‐choice format was chosen to closely replicate other assessments throughout the curriculum at King's College London as well as ease accessibility. There was no time limit for the tests. Students were informed of their scores (number of questions answered correctly) immediately after submission, with the opportunity to review feedback on incorrect answers retrospectively. To ensure data integrity and validity, only students who have completed both Test 1 and Test 2 were included in the study, and only first attempts were recorded.

### Analysis of test scores

Mean scores were compared between Test 1 (before anatomy teaching) and Test 2 (after anatomy teaching) using the paired *t*‐test (two‐tailed). This includes overall mean scores, mean scores of SBST vs. anatomical questions, and mean scores of anatomical questions excluding questions related to the abdomen and pelvis. The justification for observing results excluding abdomen and pelvis questions was to assess whether changes in performance extended to anatomical structures that have not been explicitly taught (i.e., regions outside the abdomen and pelvis). Finally, overall mean scores were compared between male and female students using the chi‐squared test to investigate gender difference in spatial performance. Bonferroni correction was applied to *p*‐values of significance to account for multiple comparisons. Analysis was performed using the SPSS Statistics Software (IBM, version 27).

## RESULTS

In this study, overall mean scores, mean scores of anatomy questions, and SBST questions were compared between Test 1 (before anatomy teaching) and Test 2 (after anatomy teaching). Detailed score distributions in each cohort are provided in Figures S1 and S2. Cronbach's alpha was low across all cohorts (<0.600), suggesting a low internal consistency and large variation of responses across questions (Table S3). However, Test 2 (post‐anatomy teaching) showed a higher Cronbach's alpha compared to Test 1 (pre‐anatomy teaching) in all cohorts.

### Overall mean scores were reduced after anatomy teaching

**FIGURE 3:**
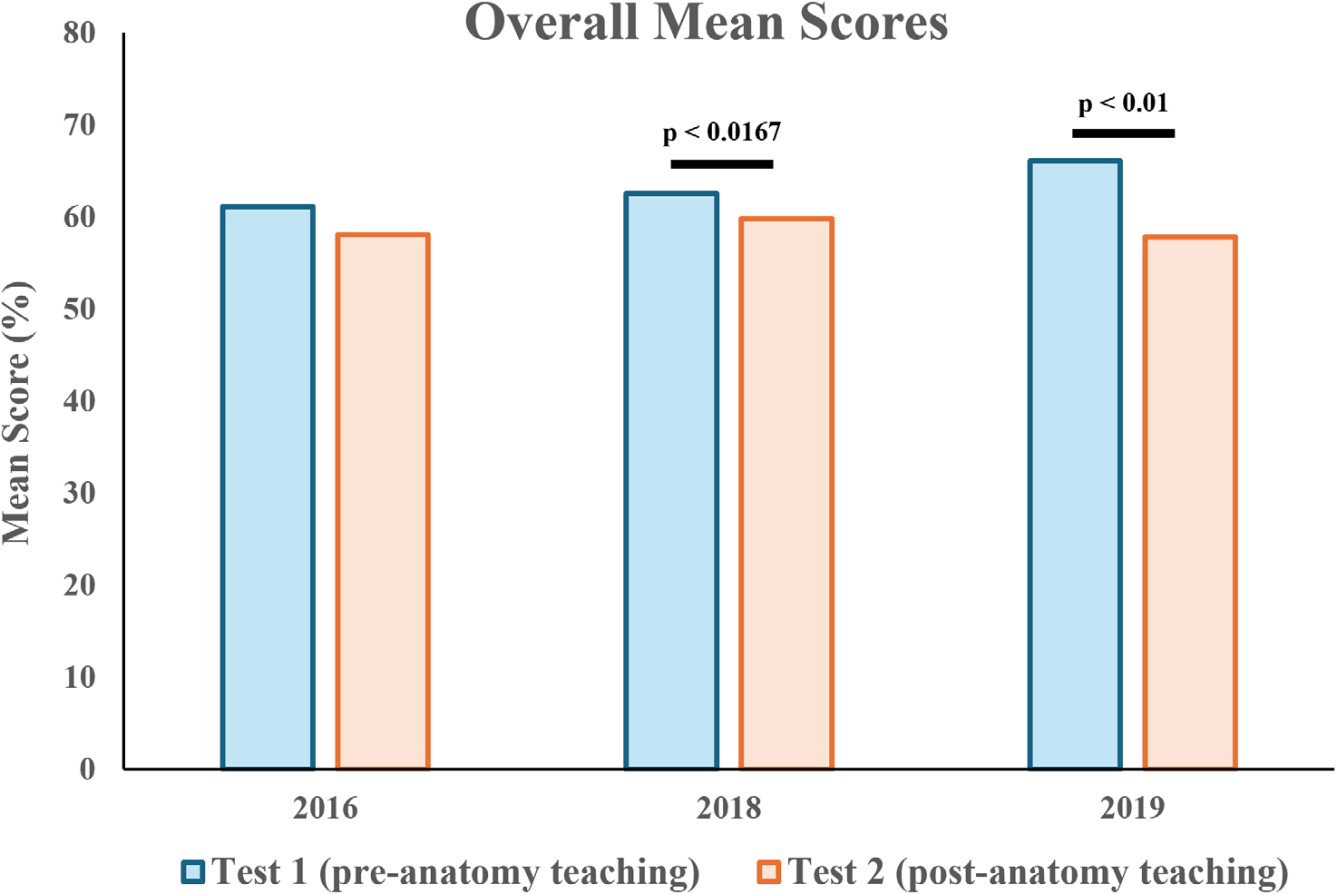
Overall mean scores between Test 1 (pre‐anatomy teaching) and Test 2 (post‐anatomy teaching). A statistically significant decrease in mean scores was observed in the 2018 and 2019 cohorts.

Surprisingly, overall mean scores were significantly decreased in the 2018 and 2019 cohorts after anatomy teaching (mean 2018: 62.5% vs. 59.8%; 2019: 66.1% vs. 57.8%) (Figure 3). A decrease was also observed in the 2016 cohort (61.1% vs. 58.1%), but was not statistically significant.

### Mean scores of anatomical questions excluding the abdomen and pelvis were increased after anatomy teaching

**FIGURE 4:**
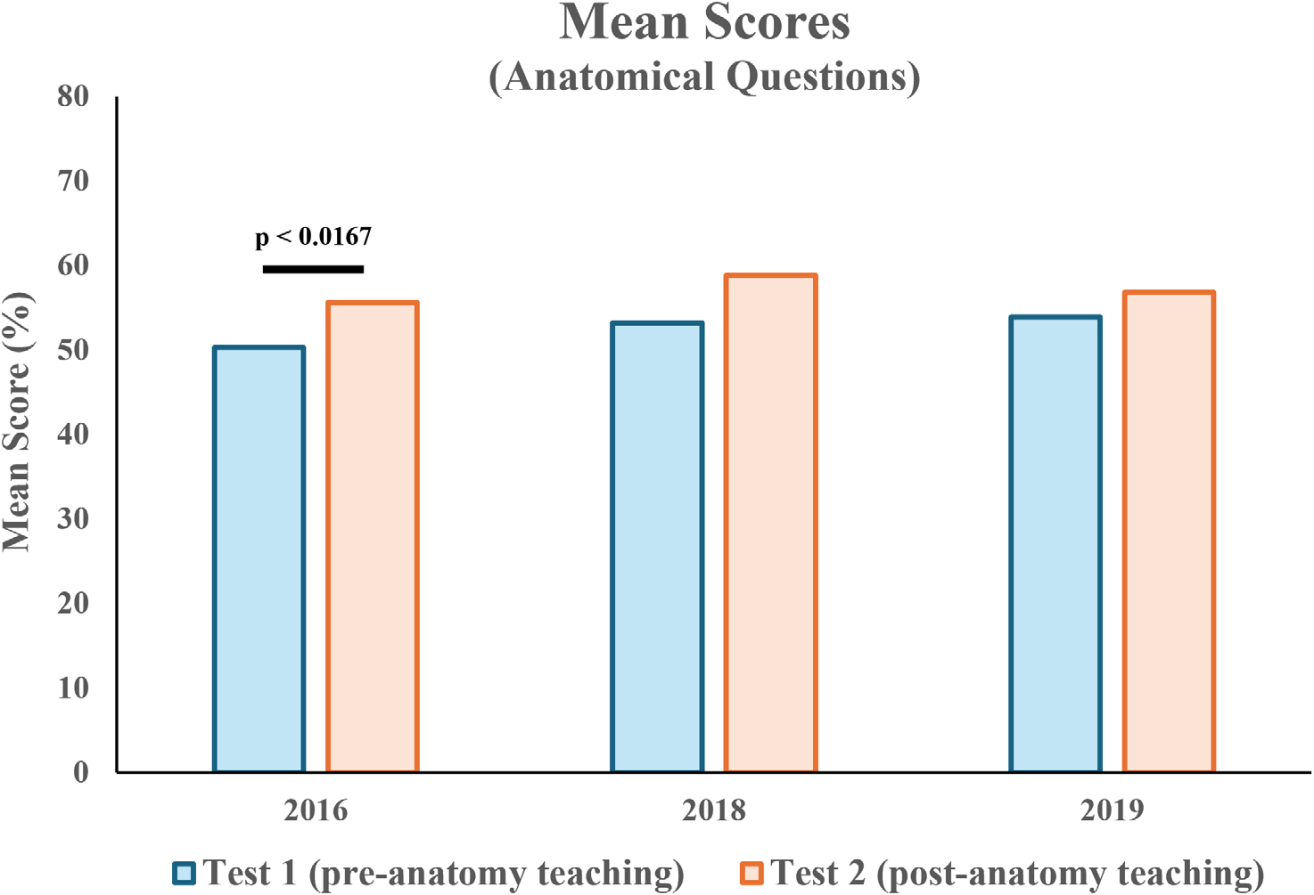
Mean scores of anatomical questions between Test 1 and Test 2. A statistically significant increase was observed in the 2016 cohort.

For anatomy questions, mean scores increased significantly for the 2016 cohort (mean 50.3% vs. 55.6%) (Figure 4). While an increase was also observed for the 2018 and 2019 cohorts (mean 2018: 53.2% vs. 58.8%; 2019: 53.9% vs. 56.8%), it was not statistically significant.

**FIGURE 5:**
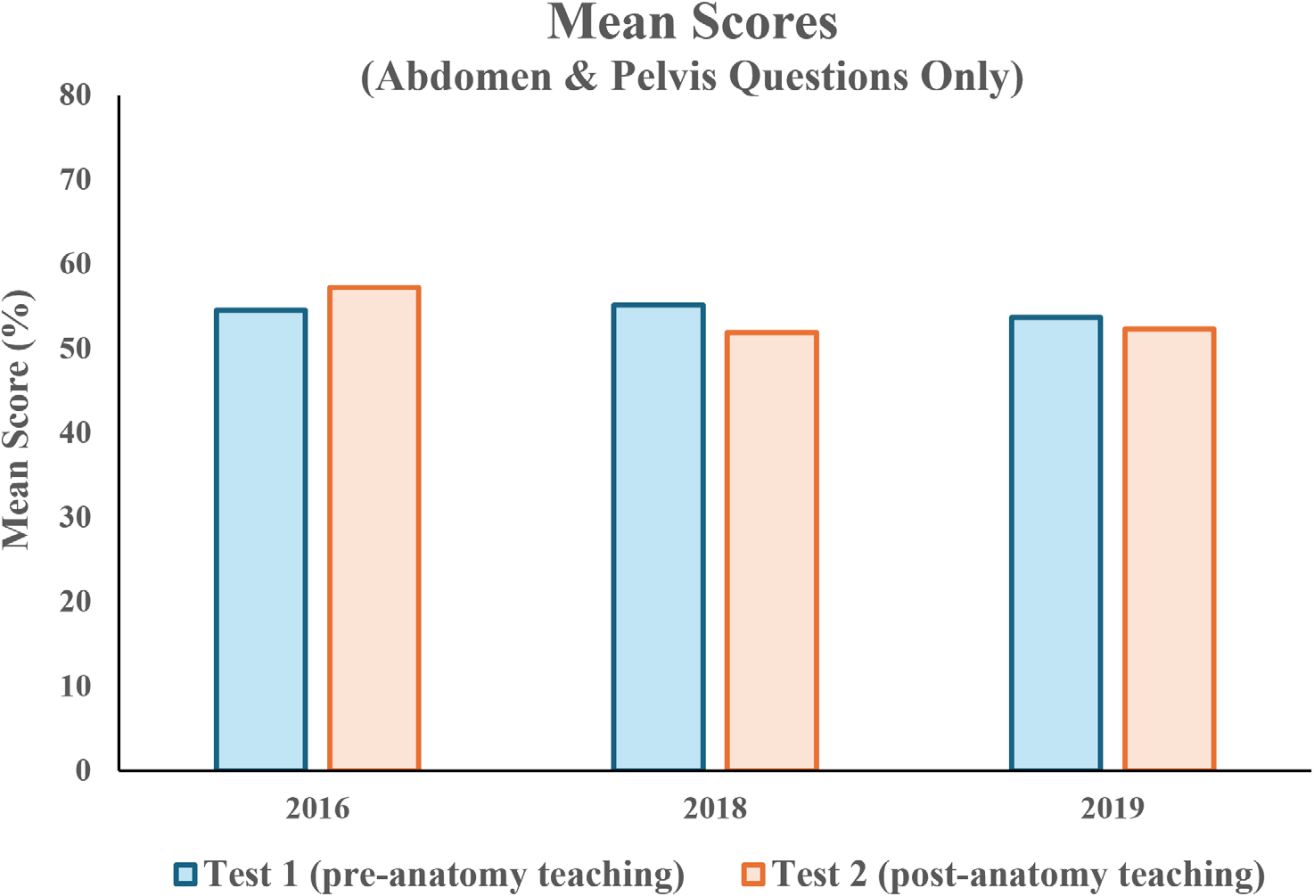
No significant difference was observed in mean scores of abdomen and pelvis questions between Test 1 and Test 2.

**FIGURE 6:**
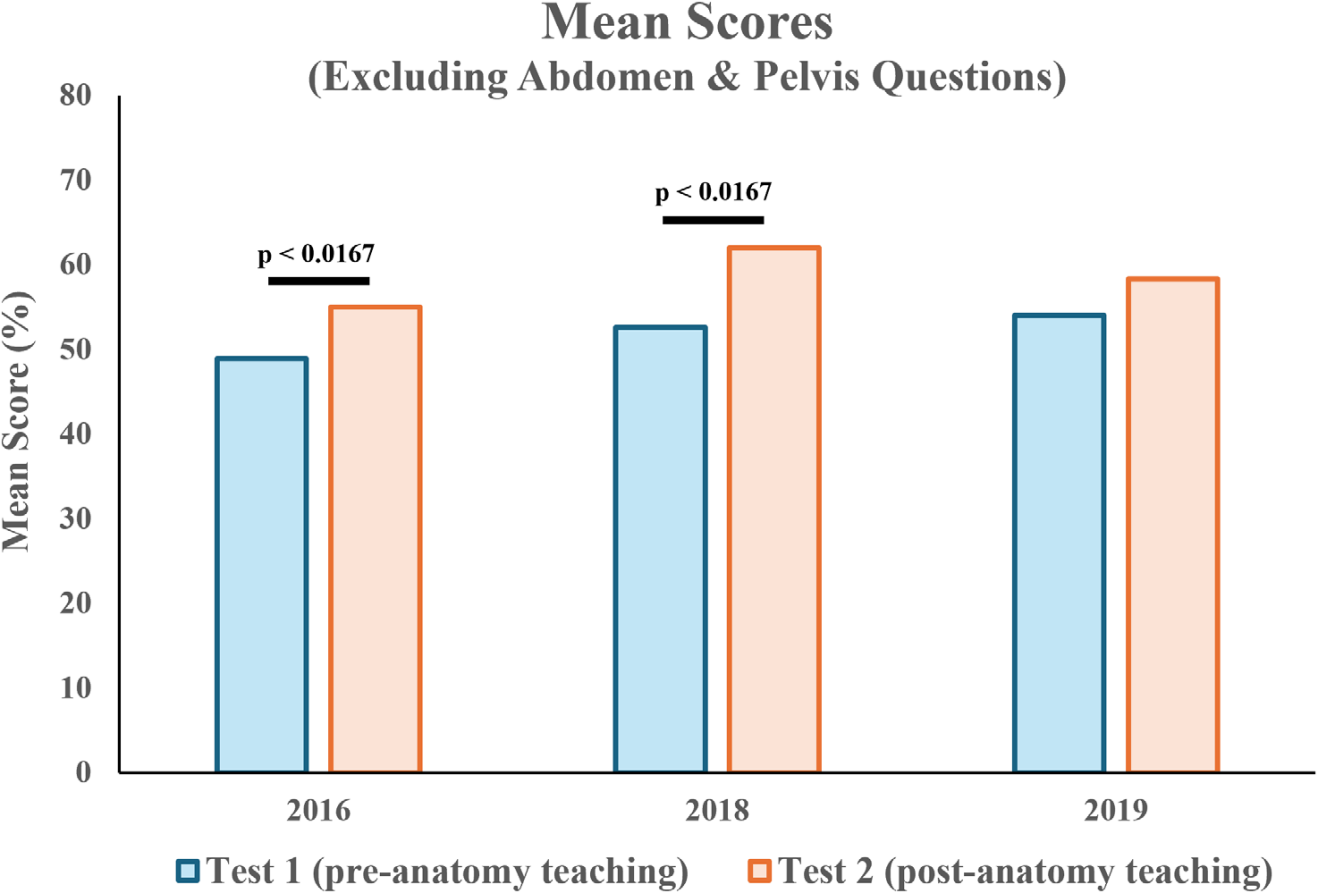
Mean scores of anatomical questions excluding the abdomen and pelvis, between Test 1 and Test 2. A significant increase was observed in 2016 and 2018 cohorts.

Considering abdomen and pelvis questions only, no significant difference was observed in mean scores between Tests 1 and 2 across all cohorts (mean 2016: 54.5% vs. 57.2%; 2018: 55.1% vs. 51.9%; 2019: 53.7% vs. 52.3%) (Figure 5). For anatomical questions excluding the abdomen and pelvis, mean scores increased significantly in 2016 and 2018 cohorts (mean 2016: 48.9% vs. 55.0%; 2018: 52.6% vs. 62.0%) (Figure 6). An increase was also observed in the 2019 cohort (54.0% vs. 58.3%), but was not statistically significant.

### Mean scores of SBST questions were reduced after anatomy teaching

**FIGURE 7:**
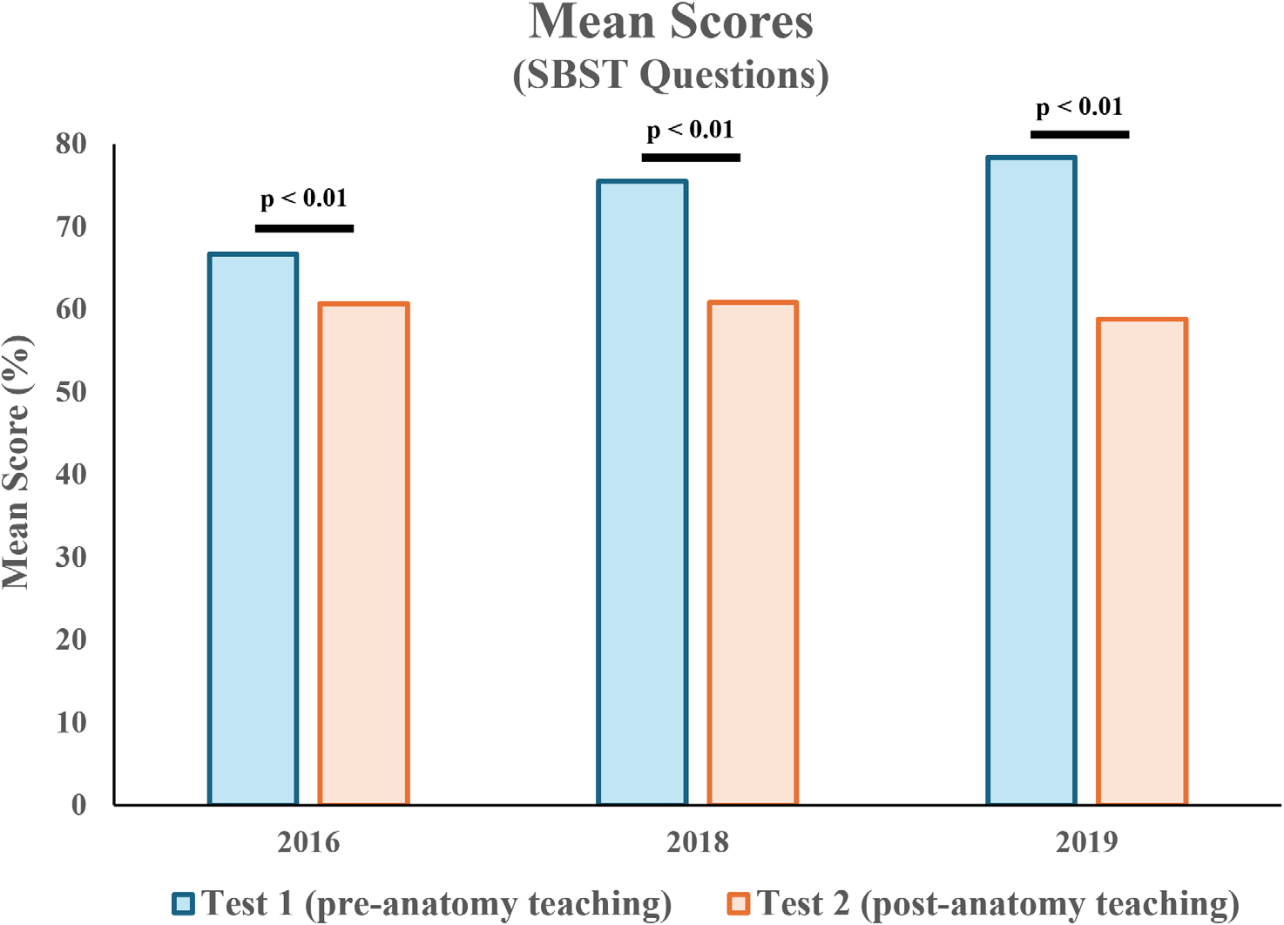
Mean scores of SBST questions between Test 1 and Test 2. A significant decrease was observed across all cohorts.

For SBST questions, a significant decrease was observed across all three cohorts (mean 2016: 66.7% vs. 60.7%; 2018: 75.5% vs. 60.8%; 2019: 78.4% vs. 58.8%) (Figure 7).

### No significant difference in spatial performance was observed between males and females

**FIGURE 8:**
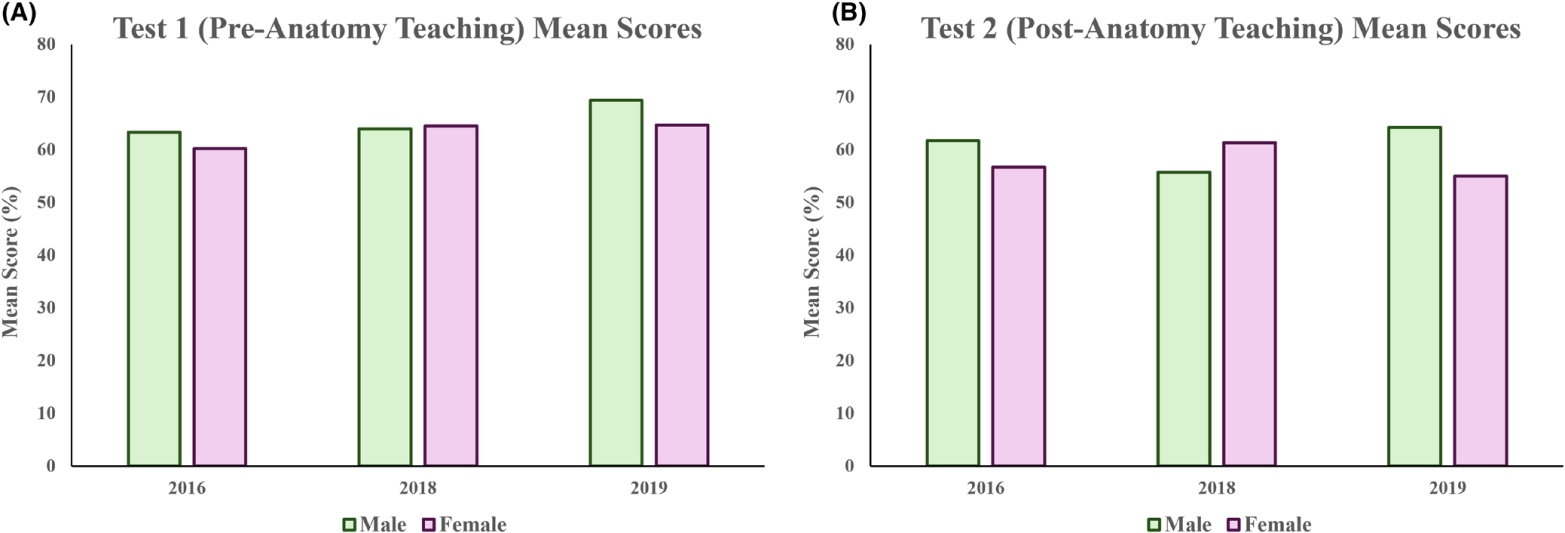
No significant difference was observed in overall mean scores between male and female students for Test 1 (A) and Test 2 (B) respectively.

Between male vs. female students, no significant difference in overall mean scores was observed in both Test 1 (mean 2016: male 63.3% vs. female 60.2%; 2018: 64.0% vs. 64.5%; 2019: 69.4% vs. 64.7%) and Test 2 (mean 2016: 61.7% vs. 56.7; 2018: 55.7% vs. 61.3%; 2019: 64.2% vs. 55.0%) across all cohorts (Figure 8).

## DISCUSSION

### Spatial reasoning of anatomical structures may be malleable through anatomy teaching

This study adds to the growing body of literature exploring whether the spatial reasoning of anatomical structures and standard geometric objects could be improved over time through anatomical teaching. Using two bespoke online tests, the study aims to determine the effect of anatomy teaching on medical students' ability to identify cross‐sectional images of geometric solids (SBST questions) and anatomical structures (anatomical questions) through comparing their mean scores between pre‐anatomy teaching (Test 1) and post‐anatomy teaching (Test 2). Results demonstrate a trend toward improved performance in anatomy questions following anatomy teaching, although the extent of improvement was not consistent across cohorts (Figure 4).

These findings suggest that anatomy teaching may have potential benefits for improving spatial reasoning of anatomical cross‐sections. This interpretation aligns with previous studies in the literature. An earlier study by Rengier, Häfner, and Unterhinninghofen in 2013, on a small group of 4th and 5th year medical students in a radiology course, demonstrated that training using interactive software led to an improvement of 38.7% in diagnostic skills from cross‐sectional images.^53^ Similarly, a study by Lufler et al.^6^ on 352 first‐year medical students demonstrated that participation in a gross anatomy course was associated with positive improvements in spatial skills.^6^

Taken together, these findings lend some support to our hypothesis that spatial ability may be malleable through anatomy teaching, consistent with Piaget's Schema theory and Paivio's Dual Coding theory. Notably, when questions relating specifically to the abdomen and pelvis (the anatomical regions taught between Test 1 and Test 2) were excluded, the trend toward improved mean scores remained evident, reaching statistical significance in 2016 and 2018 cohorts (Figure 6). This raises the possibility that anatomy teaching may positively influence students' spatial reasoning beyond the specific anatomical regions covered, which could be further explained by the development and activation process of mental schemas. Throughout anatomy teaching, schemas become increasingly detailed as students learn to recognize common patterns and configurations across various body systems; in turn, the activation of relevant schemas developed from past learning experiences allows students to analyze and interpret novel anatomical images from topics not previously taught. For example, schemas developed from studying the cardiovascular system, such as the organization of arteries and veins, may be applied to the structural understanding of nerve branches and airways. This generalization of schemas during the learning process may be a key facilitator in the transfer of spatial skills within different contexts in anatomy. Additionally, the combination of verbal and visual‐based learning during anatomy teaching, through a blend of lectures, case studies and tutor‐led dissections, may play a critical role in the construction of enriched and versatile schemas, further contributing to students' improved spatial performance across various body systems.

It is therefore encouraged that targeted strategies be incorporated to enhance spatial skills in the anatomy curriculum. In addition to fostering a blended learning environment through verbal and visual instructional techniques, novel approaches such as 3D modeling,^54^ interactive medical imaging software, and virtual or augmented reality stimulations^55^, ^56^ could be systematically integrated to provide a dynamic, multimodal learning experience. Furthermore, the transferability of spatial skills across anatomical regions suggests that teaching methodologies should focus on the development of generalized mental schemas. This could potentially be achieved through a spiral curriculum, where foundational exercises in spatial reasoning are routinely revisited and reinforced with progressively complex anatomical images across multiple body systems. Finally, incorporating assessments that specifically measure spatial reasoning could help educators identify students who may benefit from additional support.^57^

### Improvements in spatial reasoning through anatomy teaching may notransferablerable to geometric objects

The study also observed a significant decrease in performance in SBST questions across all three cohorts, which was unexpected (Figure 7). This suggests that spatial skills developed in the context of anatomy may not be transferable to the broader domain of geometric solids. While the focused approach of regional anatomy teaching may have led students to develop expertise in analyzing intricate anatomical structures, there appear to be gaps that need addressing to enable the transfer of spatial ability to more general and abstract tasks. These findings resonate with the study by Heil et al. exploring the effect of performance in mental rotation tasks before and after repetitive training, suggesting that certain aspects of spatial skills were not transferable to new objects and their varied perspectives.^58^

### Gender and spatial performance

Gender differences have long been studied in the field of spatial ability. While studies in the literature have reported mixed findings, emerging evidence has suggested that males tend to outperform females in spatial ability, particularly in mental rotation tasks.^38^, ^39^, ^40^, ^41^, ^42^ Nevertheless, there remains a paucity of studies investigating gender differences in other facets of spatial ability such as spatial perception and visualization. Meta‐analyses by Linn and Petersen^2^ and Voyer et al.^59^ reported that while males outperformed females in mental rotation and spatial perception, no significant differences were observed for spatial visualization tasks, suggesting that gender differences may vary between different aspects of spatial ability. Furthermore, whether this gender disparity is innate or can be changed over time through formal or informal training remains unclear.

In this study, no significant difference in spatial ability was observed between male and female students in the analysis of cross‐sectional images, both before and after anatomy teaching (Figure 8). The fact that all participants, being first‐year medical students, have demonstrated similar academic competence upon entry to medical school suggests that gender may not have influenced spatial ability as much as previously assumed; this uniformity in initial academic performance implies that both male and female students possess comparable spatial skills at the outset of their medical education. Future longitudinal studies may offer insight into how spatial skills evolve throughout anatomy education in the long term, and whether specific learning activities such as dissections, clinical imaging demonstrations, and virtual 3D models can enhance performance in different aspects of spatial ability. Additionally, exploring the variations in students' educational backgrounds and prior experience with spatial reasoning tasks could further elucidate how environmental factors and innate ability influence the effectiveness of anatomy teaching at improving spatial performance.

### Limitations

This study has several limitations. Firstly, the trends observed in this study were statistically significant only for some, but not all, student cohorts. This may be due to the limited number of questions in both tests, as well as the limited sample size of student participants, possibly because the tests were non‐mandatory and formative in nature. Therefore, while the study observed a trend that spatial skills may be malleable through anatomy teaching, further longitudinal studies involving larger sample sizes are warranted.

Secondly, Cronbach's alpha values were low across all cohorts (<0.600), suggesting limited internal consistency of test responses. This is likely due to the heterogeneity of topics and question types. Anatomy questions covering different body regions may not correlate strongly with one another, and the inclusion of items involving standard geometric objects, unrelated to anatomical content, may further reduce consistency. In future studies, a factor analysis may help determine whether the test items group into distinct dimensions. Additionally, variability in students' learning progress may play a role. In Test 1, students are still in the early stages of anatomy education, often relying on fragmented knowledge and guesswork. By Test 2, i.e., two months into their first‐year education, they are expected to have encountered more anatomical material, leading to more structured and integrated knowledge. This may account for the slight improvement in Cronbach's alpha scores in Test 2 compared to Test 1 across all three cohorts.

Lastly, although the content of anatomy teaching during the interventional period has remained consistent across the three cohorts, the way in which students revised the teaching material and the amount of time spent on learning anatomy outside of teaching hours, such as through self‐guided online materials, is neither quantifiable nor controllable. It would therefore be of interest to investigate the extent of students' exposure to anatomy learning beyond the curriculum and its association with improvements in spatial performance. This may be addressed in future studies.

## CONCLUSION

This study is one of the first to explore the potential malleability of spatial skills through anatomy teaching among medical students, in the context of interpreting cross‐sectional images of anatomical structures and geometrical objects. Findings suggest that spatial skills may be malleable and enhanced through anatomy teaching. Furthermore, improvements in spatial ability may be transferable across body systems through the development and activation of mental schemas, suggesting a broader applicability in anatomy education that may allow students to apply their enhanced skills in new anatomical contexts. However, there is no significant evidence of its transferability to broader domains such as abstract geometrical objects, indicating that such improvements are context‐specific and may not generalize to all types of spatial reasoning tasks. Finally, the study identifies no significant correlation between gender and spatial ability, both before and after anatomy education.

## AUTHOR CONTRIBUTIONS

**Mandeep Gill Sagoo:** Conceptualization; data curation; investigation; methodology; project administration; resources; supervision; validation; writing – review and editing. **Pak Yin Lam:** Data curation; formal analysis; investigation; methodology; validation; writing – original draft. **Sharukesi Theivendran:** Data curation; formal analysis; methodology; validation; writing – original draft; writing – review and editing. **Richard Wingate:** Conceptualization; data curation; resources.

## ETHICS STATEMENT

Ethical approval was first obtained from the King's Research Ethics Committee on 20th July 2016, in compliance with the General Data Protection Regulations (reference no. LRS‐15/16‐3391). It was subsequently renewed in 2018 (reference no. LRS‐17/18‐8318) and in 2019 (reference no. LRS‐19/20‐8318). Informed consent was obtained from student participants through an online form prior to the study.

## Supporting information


**Supplementary Table 1.** *Functional Anatomy and Embryology* module curriculum for first‐year medical students at King’s College London between 2016–2020. The module runs throughout the first term, from end of September to mid‐December. It covers the basics of cell and tissue organization; muscles, nerves, skeleton and joints; anatomy of the abdomen and pelvis, including gastrointestinal, hepatobiliary, urinary and reproductive systems; and phases of embryological development.
**Supplementary Table 2**. Time spent per week for each anatomy teaching activity.
**Supplementary Table 3**. Cronbach’s alpha calculations for each cohort.
**Supplementary Figure 1**. Distributions of overall mean scores in Test 1 (pre‐anatomy teaching) and Test 2 (post‐anatomy teaching) in each yearly cohort. All cohorts are shown to follow a normal distribution.
**Supplementary Figure 2**. Distributions of overall mean scores, mean scores for anatomical questions, and mean scores for SBST questions.
