# Supplementary material for: Exploring the potential malleability of spatial skills through anatomy teaching: A quantitative study among medical students
Source: Anat Sci Educ. 2025 Jun 13;18(9):961–71. doi: 10.1002/ase.70071 (PMC12413475; doi:10.1002/ase.70071)

**Supplementary Table 1.** *Functional Anatomy and Embryology* module curriculum for first-year medical students at King’s College London between 2016−2020. The module runs throughout the first term, from end of September to mid-December. It covers the basics of cell and tissue organization; muscles, nerves, skeleton and joints; anatomy of the abdomen and pelvis, including gastrointestinal, hepatobiliary, urinary and reproductive systems; and phases of embryological development.

| **Month** | **Topic of anatomy teaching** | **Teaching activities** | **Total hours** |
| --- | --- | --- | --- |
| End of September | Anatomical terminology | Lectures, workshops, dissections | 18 |
|  | Cell & tissue organization and imaging technologies |  |  |
| October − November |  |  |  |
|  | Overview of the skeleton, joints and peripheral nervous system |  | 17 |
|  | Abdominal wall anatomy |  | 12 |
|  | Stomach, spleen, hepatobiliary system and anal canal |  | 18 |
|  | Retroperitoneal organs and urinary system |  | 8 |
| December | Pelvis and perineum anatomy |  | 15 |
|  | Male and female reproductive systems |  |  |
|  | Embryological development | Lectures | 2 |

**Supplementary Table 2.** Time spent per week for each anatomy teaching activity.

| **Teaching activity** | **Hours per week** | **Description** |
| --- | --- | --- |
| Lectures | 4−6 | Oral presentations given by professors and lecturers from the Anatomy Department, delivered to the entire student cohort. Contents are presented through PowerPoint, and include schematic diagrams of anatomical regions, as well as images and videos of cadavers and clinical scans. |
| Workshops | 2−4 | Small-group teaching sessions involving groups of 5−10 students, facilitated by a member of teaching staff, usually a tutor or senior researcher. Students revise and discuss topics covered in recent lectures, with occasional demonstrations of histological slides, 3D models, and bedside clinical imaging workshops such as ultrasound scans. There are 1−2 sessions per week, each lasting 2 hours. |
| Dissections | 2−4 | Students divide into small groups of 5−10 to take part in cadaveric dissections. Each group is assigned an anatomy demonstrator to guide the dissection process and explain the relevant anatomy. There are 1−2 dissections per week, each lasting 2 hours. Each session focuses on a specific anatomical region, e.g. the abdominal wall, the peritoneum and inguinal region, the pelvis and perineum etc. |

**Supplementary Table 3.** Cronbach’s alpha calculations for each cohort.

| **Year** | **Test** | **Cronbach’s Alpha** | **Number of Questions** |
| --- | --- | --- | --- |
| 2016 | Test 1 | 0.504 | 24 |
|  | Test 2 | 0.516 | 24 |
| 2018 | Test 1 | 0.350 | 24 |
|  | Test 2 | 0.577 | 24 |
| 2019 | Test 1 | 0.516 | 24 |
|  | Test 2 | 0.584 | 24 |

**Supplementary Figure 1.** Distributions of overall mean scores in Test 1 (pre-anatomy teaching) and Test 2 (post-anatomy teaching) in each yearly cohort. All cohorts are shown to follow a normal distribution.

**
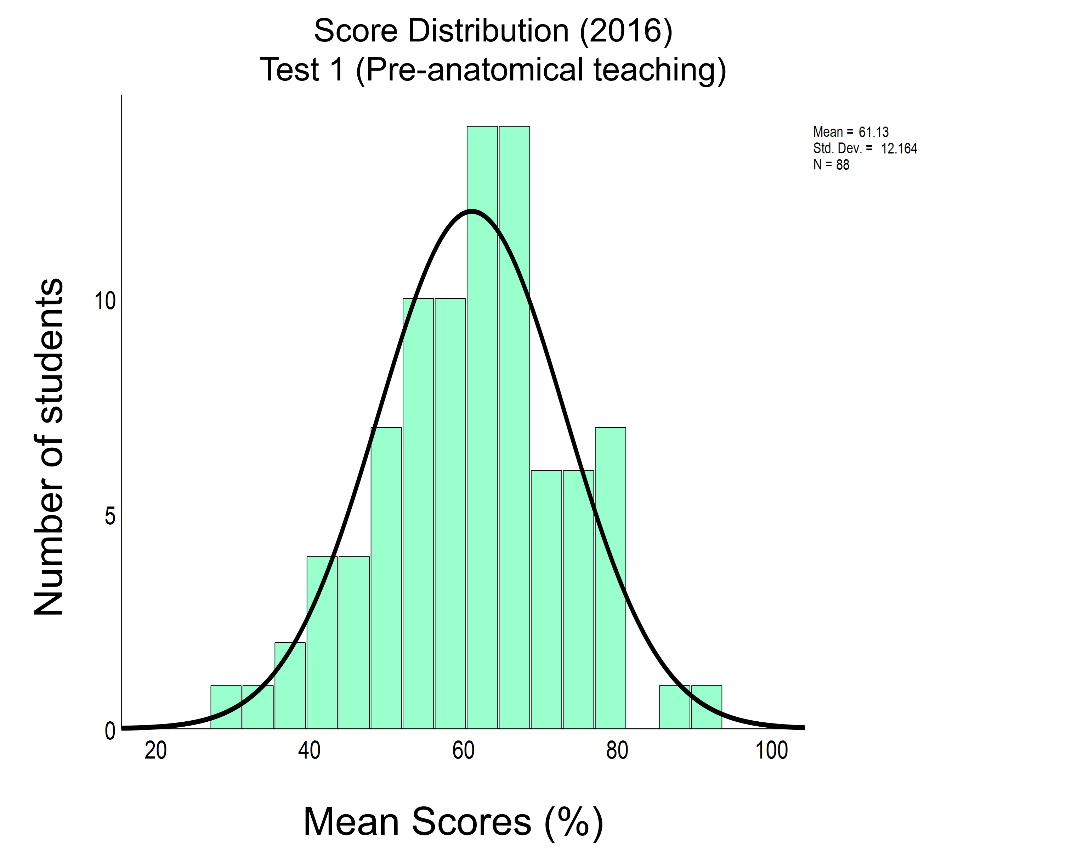
**


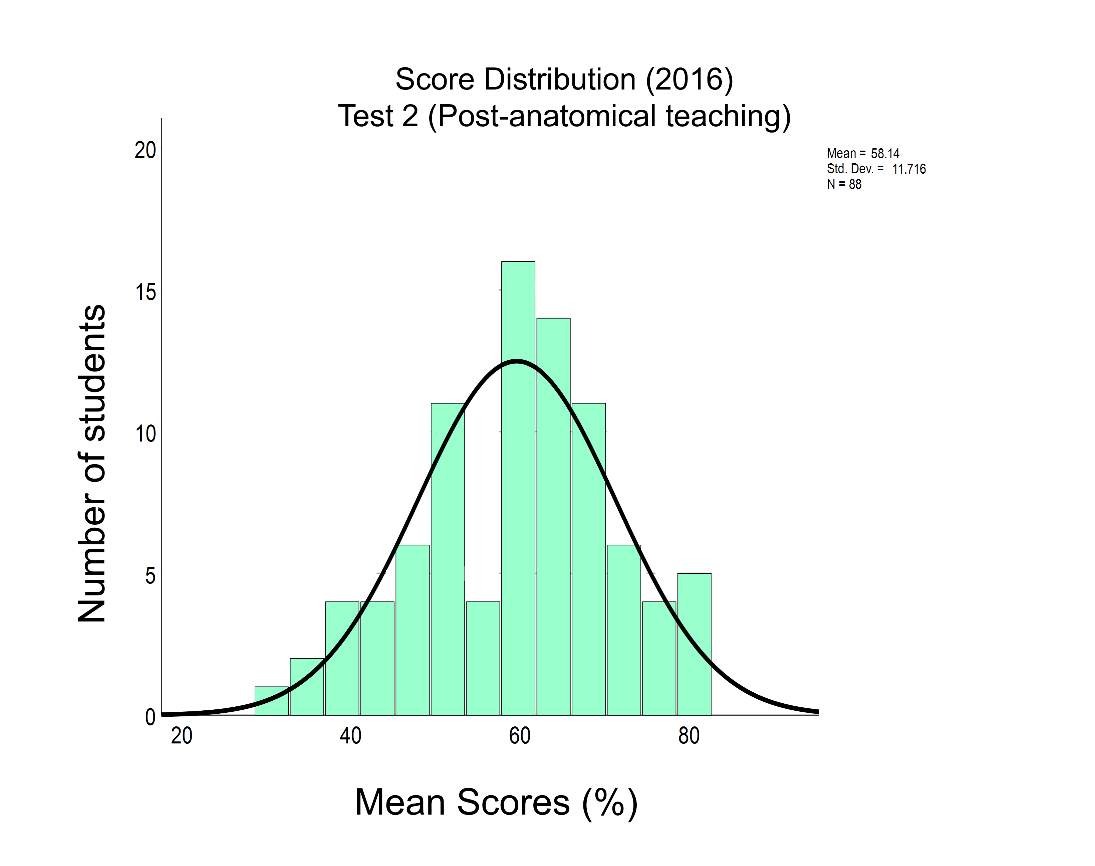


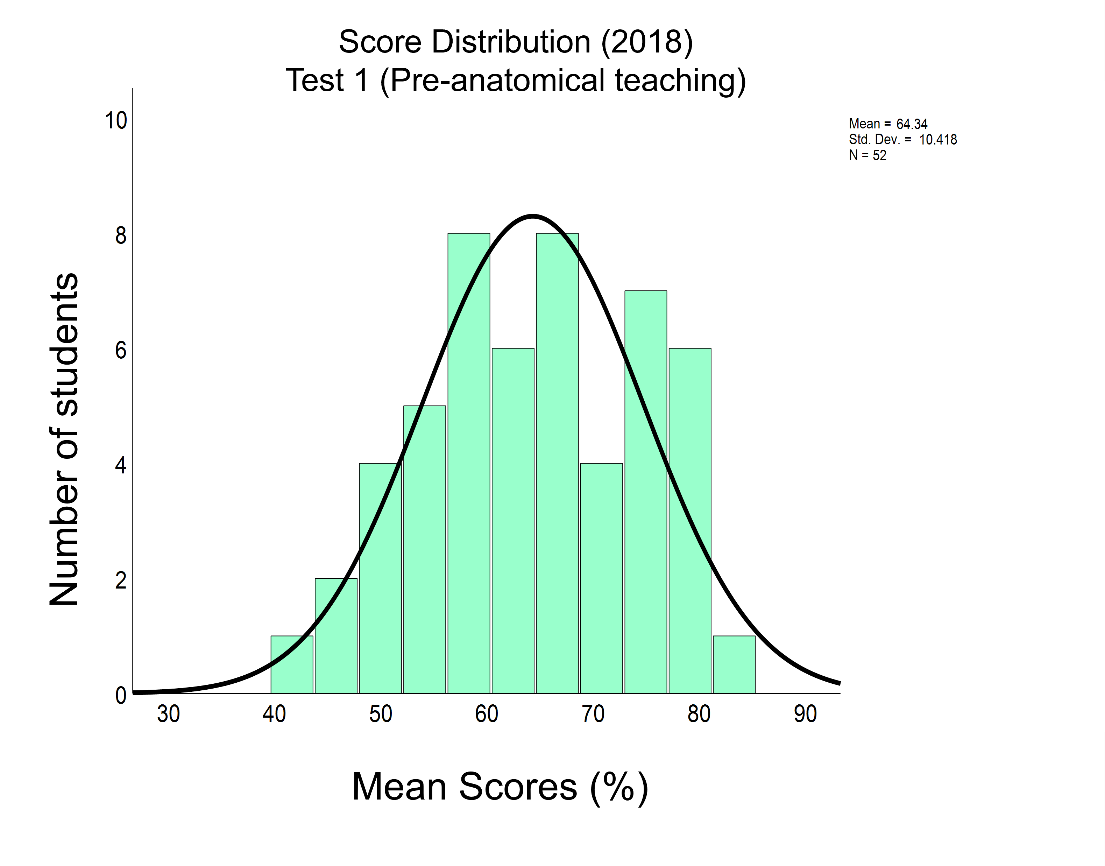


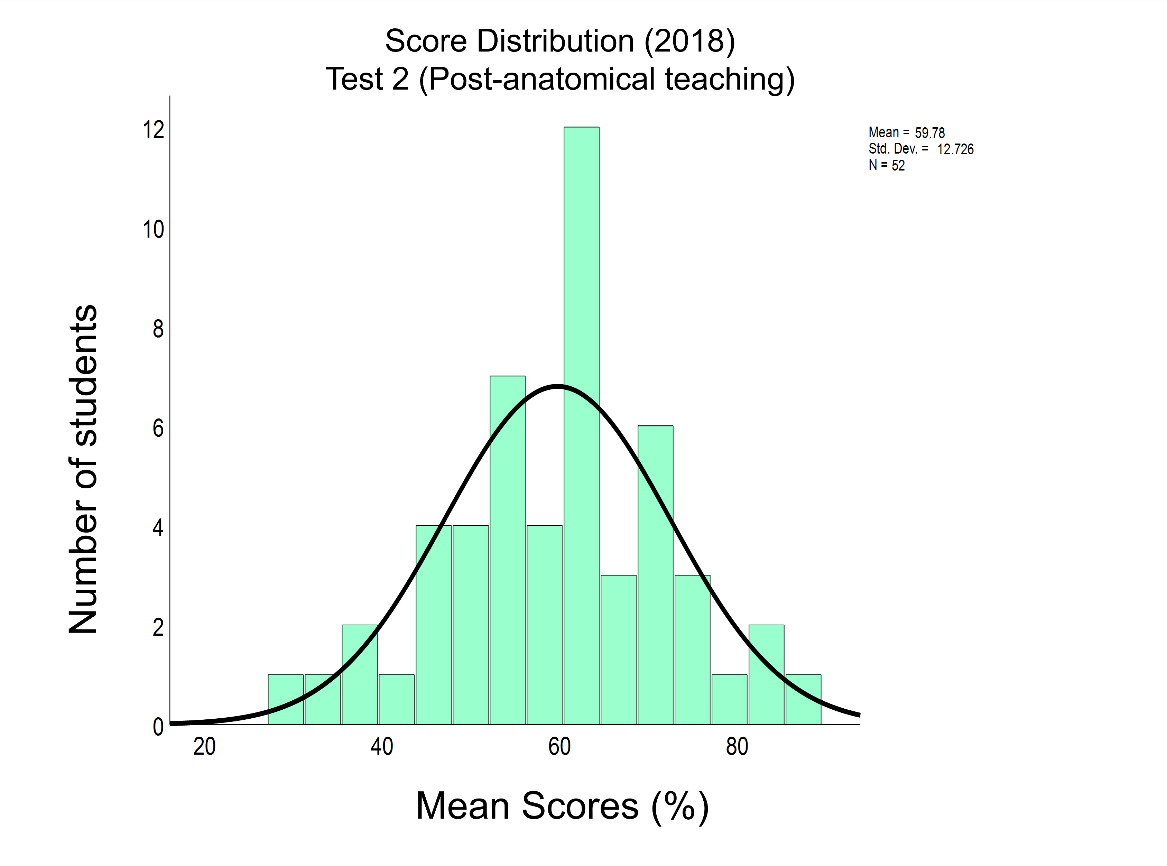


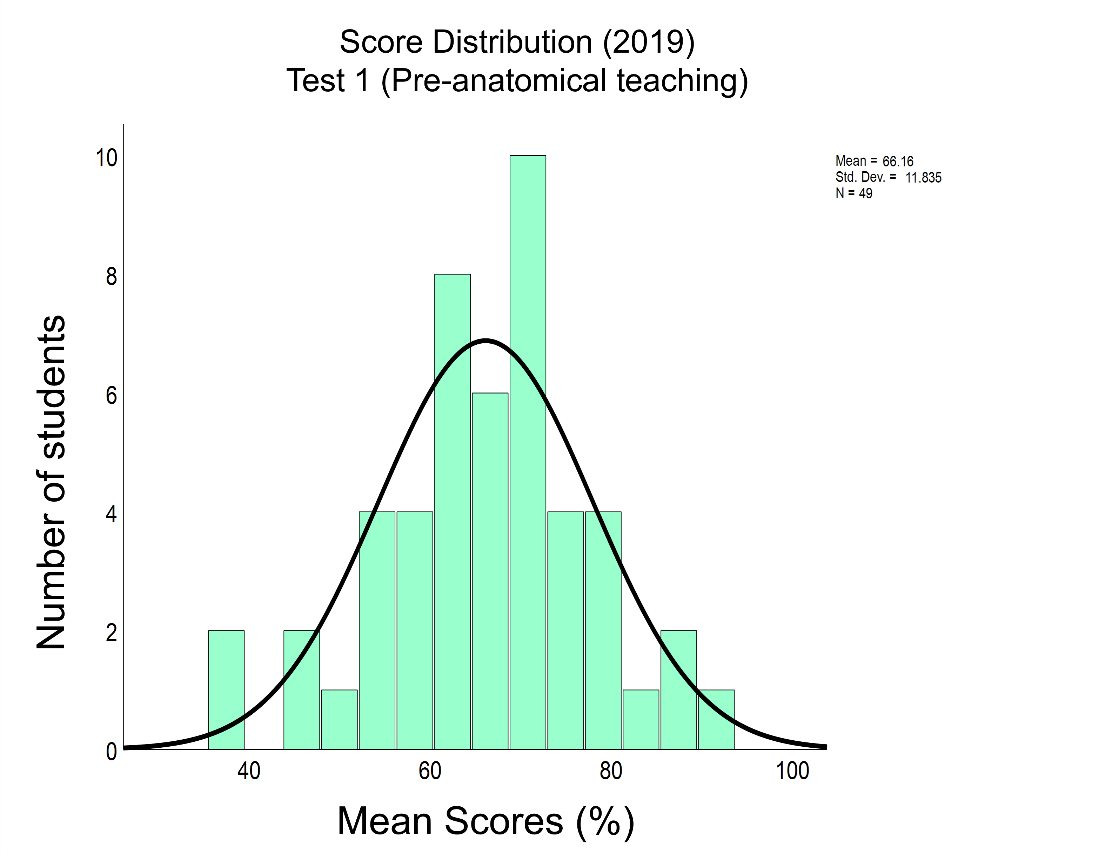


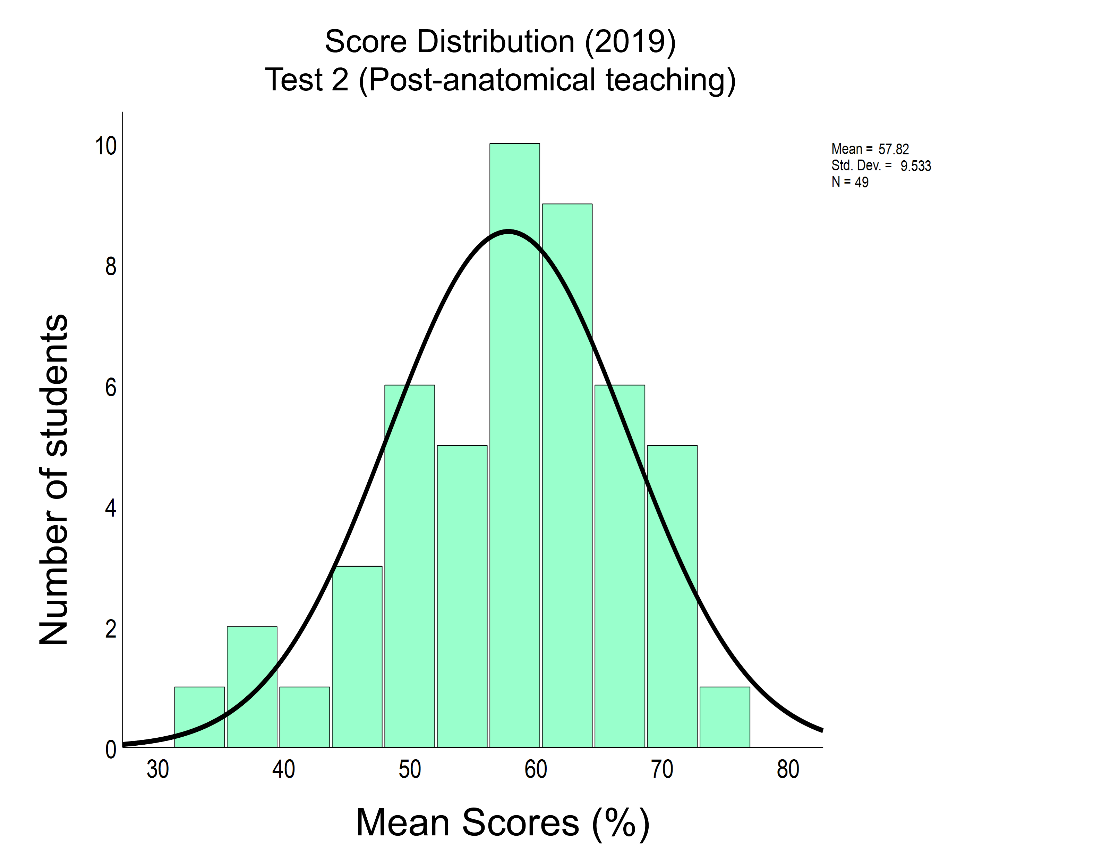


**Supplementary Figure 2.** Distributions of overall mean scores, mean scores for anatomical questions, and mean scores for SBST questions.


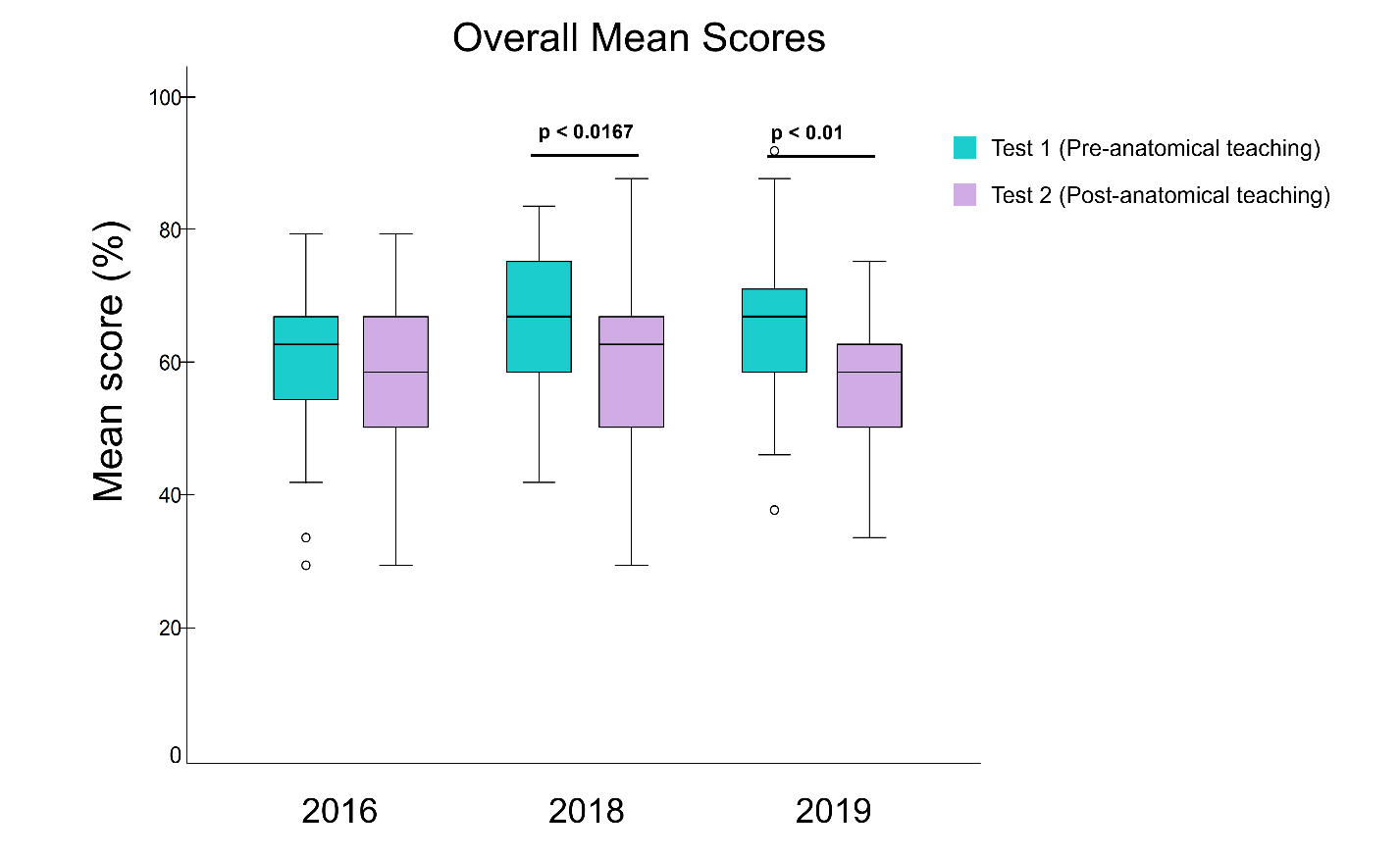


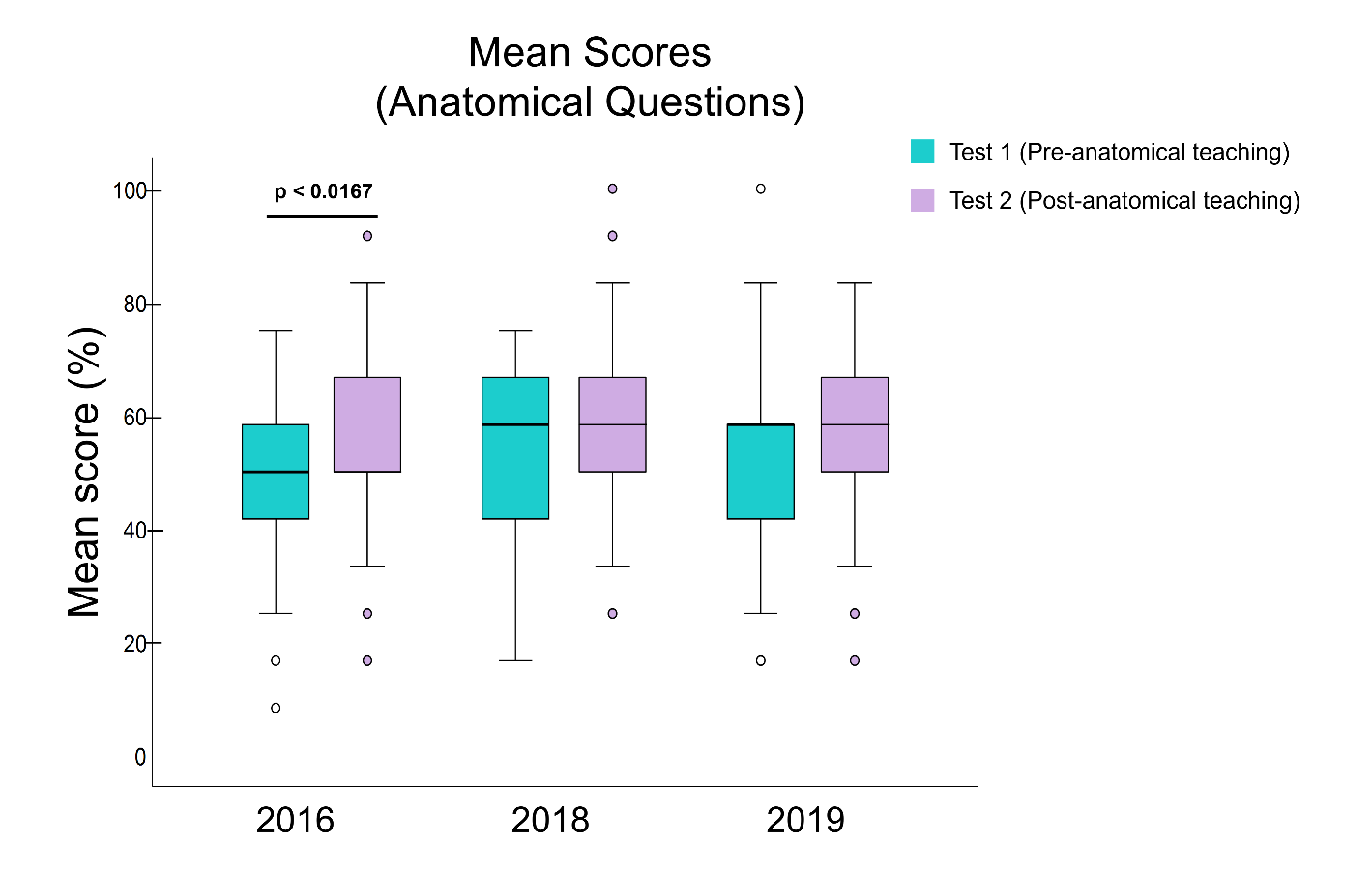


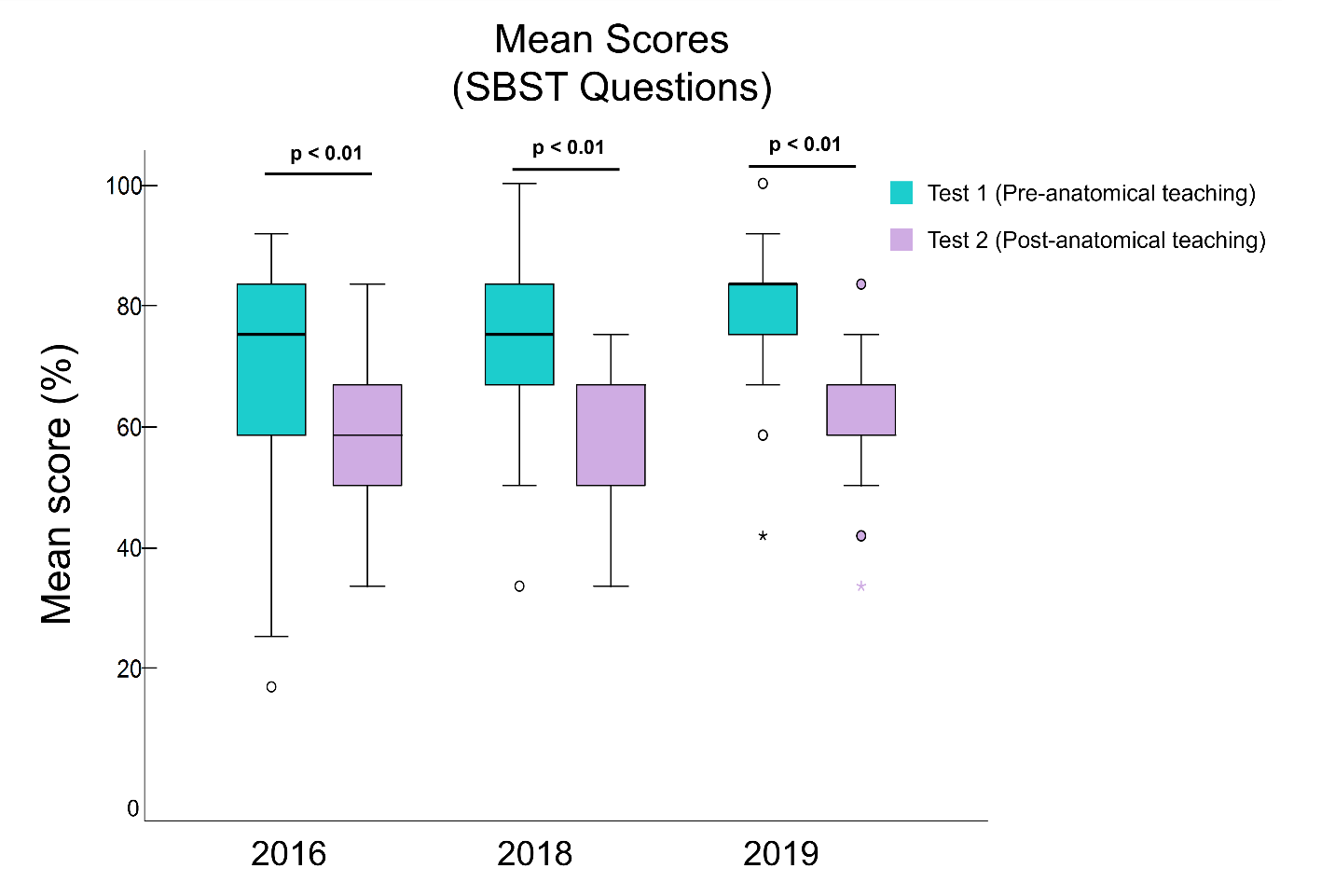

Supplement: Supplementary file 1 — Supplementary Table 1. Functional Anatomy and Embryology module curriculum for first‐year medical students at King’s College London between 2016–2020. The module runs throughout the first term, from end of September to mid‐December. It covers the basics of cell and tissue organization; muscles, nerves, skeleton and joints; anatomy of the abdomen and pelvis, including gastrointestinal, hepatobiliary, urinary and reproductive systems; and phases of embryological development. Supplementary Table 2. Time spent per week for each anatomy teaching activity. Supplementary Table 3. Cronbach’s alpha calculations for each cohort. Supplementary Figure 1. Distributions of overall mean scores in Test 1 (pre‐anatomy teaching) and Test 2 (post‐anatomy teaching) in each yearly cohort. All cohorts are shown to follow a normal distribution. Supplementary Figure 2. Distributions of overall mean scores, mean scores for anatomical questions, and mean scores for SBST questions. [file ASE-18-961-s001.docx]
